# Associations of IL-27 Polymorphisms and Serum IL-27p28 Levels With Osteosarcoma Risk

**DOI:** 10.1097/MD.0000000000000056

**Published:** 2014-08-22

**Authors:** Yu-jin Tang, Jun-li Wang, Le-gen Nong, Chang-gong Lan, Zhen-gang Zha, Pin-hu Liao

**Affiliations:** First Affiliated Hospital of Jinan University, Guangzhou (Y-JT, Z-GZ, P-HL); Center of Clinical Laboratory (Y-JT, C-GL, P-HL); and Affiliated Hospital of Youjiang Medical College for Nationalities (J-LW, L-GN), Baise, Guangxi, China.

## Abstract

Interleukin (IL)-27 is a novel cytokine secreted by stimulation of antigen-presenting cells. No previous studies currently reported the role of IL-27 in the carcinogenesis of osteosarcoma. We aimed to investigate the association of IL-27 polymorphisms and serum IL-27p28 with osteosarcoma risk in a Chinese population.

One hundred and sixty osteosarcoma patients and 250 health controls were selected. IL-27 gene -964 A/G, 2905 T/G, and 4730 T/C polymorphisms were determined by using polymerase chain reaction-restriction fragment length polymorphism. Enzyme-linked immunosorbent assay were used to detect serum IL-27p28 levels.

The serum IL-27p28 levels were significantly lower in osteosarcoma patients compared with those in controls (*P *< 0.01). Serum IL-27p28 levels in stages III–IV were lower than those in stages I–II of osteosarcoma (*P* < 0.05); similar results were also found in patients with metastasis, that is, patients with metastasis have higher IL-27p28 levels than those without metastasis (*P* < 0.05). There were no associations between genotype and allele frequencies of IL-27 -964 A/G, 2905 T/G, 4730 T/C, and the risk of osteosarcoma (*P* > 0.05). Stratification analysis also failed to show the associations between -964 A/G, 2905 T/G, and 4730 T/C polymorphisms and the clinical stage and metastasis of osteosarcoma (*P* > 0.05). Three possible haplotypes (A^-964^T^2905^T^4730^, G^-964^T^2905^T^4730^, and G^-964^G^2905^C^4730^) were identified, but no associations were found between them and the osteosarcoma risk (*P* > 0.05).

This study indicates that the lower serum IL-27p28 levels may be associated with development and progression of osteosarcoma, but IL-27 gene -964 A/G, 2905 T/G, and 4730 T/C polymorphisms and their haplotypes are not associated with osteosarcoma risk.

## INTRODUCTION

Osteosarcoma is an aggressive malignant neoplasm arising from primitive transformed cells of mesenchymal. It is the most common histological form of primary bone cancer and mostly occurs in adolescents and people over 50 years of age.^1^ Osteosarcoma is featured by the differentiation of osteoblastic and production of malignant osteoid.^2^ The exact etiology of osteosarcoma remains to be elucidated. For many years, accumulated evidences suggested that multiple genetic and environmental factors play pivotal roles in the pathogenesis of osteosarcoma.^3^ Currently, there are studies indicating that the main mechanism of immune defense system against cancer is dependent on T cell response.^4^ Therefore, the polymorphisms of genes encoding T cell response molecules may potentially affect the development of tumors.^5^

Interleukin (IL)-27 is a member of the same family as IL-12 and IL-23. IL-27 is involved in the process of the differentiation of Th1 lymphocytes, enhancement of the cellular type immune response, and the reciprocal inhibition of Th2 humoral immune reactions.^6^ Evidences have demonstrated that IL-27 can inhibit tumor progression through several mechanisms, regardless of tumor immunogenicity; these characters potentially make it a valuable agent in the treatment of tumors.^7^ Human IL-27 is located at chromosome 16p11 and secreted as a heterodimer that consists of the Epstein–Barr-induced gene 3 (EBI3) product and p28. EBI3 subunit has sequence homology with IL-12p40,^8^ and the heterodimeric IL-27p28 chain belongs to the family of long-chain 4-helix bundle cytokines and displays sequence homology to IL-12p35 and IL-23p19.^9^ Recently, the associations of IL-27 -964 A/G, 2905 T/G, and 4730 T/C polymorphisms with the risk for various diseases were investigated and the relationships between -964 A/G and the risk for several diseases such as asthma,^10^ chronic obstructive pulmonary disease (COPD),^11^ and inflammatory bowel disease (IBD^12^) have been identified.

Previously, studies have investigated the association between IL-27 gene polymorphisms and several tumors, such as esophageal cancer,^13^ nasopharyngeal carcinoma,^14^ glioma,^15^ and colorectal cancer.^16^ However, little is known about the role of IL-27 gene polymorphisms in the carcinogenesis of osteosarcoma. A better understanding of the association between IL-27 gene polymorphism and osteosarcoma risk may identify an important role of IL-27 in the carcinogenesis of osteosarcoma, which provides clues that help to guide the treatment of this tumor. Therefore, to clarify this association, we analyzed the IL-27 gene -964 A/G (rs153109), 2905 T/G (rs17855750), and 4730 T/C (rs181206) and their haplotypes in osteosarcoma and normal controls in a Chinese population.

## MATERIALS AND METHODS

### Study Population

This case-control population study is hospital based and consists of 160 osteosarcoma patients and 250 healthy controls. The osteosarcoma patients were enrolled from the Affiliated Hospital of Youjiang Medical College for Nationalities and the First Affiliated Hospital of Jinan University between 2005 and 2013. The diagnosis of osteosarcoma was based on pathology test and none of the patients had familial cancer history. The healthy controls were those who visited the hospitals at the same period for general health checkup, and they have no evidences of personal or family history of cancer or other serious illness. Clinical stage of these osteosarcoma patients was classified according to the 6th edition of the tumor, node, metastasis classification of the International Union Against Cancer. All of the patients had no autoimmune diseases before their hospitalization. At the time of sample collection, none of the patients had received any anticancer therapy, corticosteroids, doxorubicin, or other nonsteroid anti-inflammatory drugs that may compromise the function of the immune system. The controls were matched with the patient population in terms of age, gender, and residence area. All participants were unrelated ethnic Han Chinese. Written informed consent was obtained from each participant. The study was approved by the Review Boards of the Affiliated Hospital of Youjiang Medical College for Nationalities.

### Serum IL-27p28 and IL-12p40 Determination

Serum samples were collected from venous blood at room temperature and stored at −80°C until use. The quantitative determination of serum IL-27p28 was performed by enzyme-linked immunosorbent assay kits (Fermentas China Co, Ltd, Shenzhen, China) according to the manufacturer’s instruction. Developed color reaction was measured as OD450 units on an enzyme-linked immunosorbent assay reader (BIO-RAD 680, Tokyo, Japan). The concentration of cytokines was determined using standard curve constructed with the kit’s standards over the range of 0–10,000 pg/mL.

### DNA Extraction

Genomic DNA was extracted from ethylene diamine tetraacetic acid anticoagulated peripheral blood leukocytes by the salting-out method. Briefly, 3 mL of blood was mixed with Triton lysis buffer (0.32 M sucrose, 1% Triton100, 5 mM MgCl_2_, H_2_O, 10 mM Tris–HCl, and pH 7.5). Leucocytes were spun down and washed with H_2_O. The pellet was incubated with proteinase K at 56°C and subsequently salted out at 4°C using a saturated NaCl solution. Precipitated proteins were removed by centrifugation. The DNA in the supernatant fluid was dissolved in 300 µL H_2_O.

### Genotype of IL-27 Variants

The IL-27 gene -964 A/G, 2905 T/G, and 4730 T/C genotypes were determined using a polymerase chain reaction (PCR)-restriction fragment length polymorphism method. The PCR primers were designed based on the GenBank reference sequence (No. EF064720) as we previously described.^14,16^ Table S1 (http://links.lww.com/MD/A33) presented primer sequences and reaction conditions. The PCR was carried out as in our previous study.^14^ To confirm the genotyping results, PCR-amplified DNA samples were examined by DNA sequencing and the results were 100% concordant.

### Statistical Analysis

Comparisons were carried out using Student’s *t*-test or χ^2^ test for 2-group comparisons when appropriate. Correlations were determined by Pearson’s correlation test. Genotype and allele frequencies of IL-27 among groups were compared by using the χ^2^ test and Fisher’s exact test when appropriate, and odds ratios (ORs) and 95% confidence intervals (CIs) were calculated to assess the relative risk conferred by a particular allele and genotype. The goodness of fit test for Hardy–Weinberg equilibrium (HWE), calculating the expected frequencies of each genotype and comparing them with the observed values for subjects, was performed using χ^2^ test. The linkage disequilibrium (LD) among the polymorphisms was quantified using the Shi’s standardized coefficient D′(|D′|). The haplotypes and their frequencies were estimated based on a Bayesian algorithm using the Phase program.^17^ Statistical significance was assumed at the *P* < 0.05 level. The SPSS statistical software package version 16.0 (SPSS, Chicago, IL) was used for all of the statistical analysis.

## RESULTS

### Clinical Characteristics of the Study Participants

The demographics and clinical characteristics of the osteosarcoma patients and controls in this study are shown in Table 1. There were no significant differences with respect to the age and gender distributions between osteosarcoma patients and controls (*P* > 0.05).

**TABLE 1:**
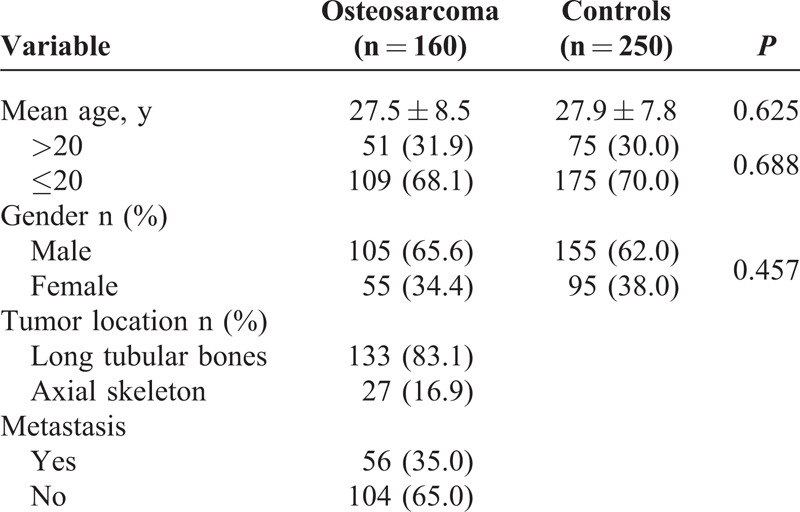
Characteristics of the Study Population

### Serum Levels of IL-27p28 and IL-12p40

The serum IL-27p28 levels were significantly elevated in healthy controls (243.7 ± 108.5 pg/mL) compared with those in osteosarcoma patients (158.6 ± 97.3 pg/mL, *P* < 0.01). We also found that serum IL-12p40 levels were distinctly elevated in healthy controls (268.2 ± 107.4 pg/mL) compared with osteosarcoma patients (201.2 ± 90.3 pg/mL, *P* < 0.01). Correlation analysis revealed that IL-27p28 was correlated with IL-12p40 (*r* = 0.567, *P* = 0.004).

### Relationship of Serum IL-27p28 and Clinical Features of Osteosarcoma

The results showed that serum IL-27p28 levels in stages III–IV were lower than those in stages I–II of osteosarcoma (*P* < 0.05); and patients with metastasis have higher IL-27p28 levels than those without metastasis (*P* < 0.05). However, no significant differences were found with respect to patients’ age, gender, tumor size, histological type, and tumor location (*P* > 0.05) (Table 2).

**TABLE 2:**
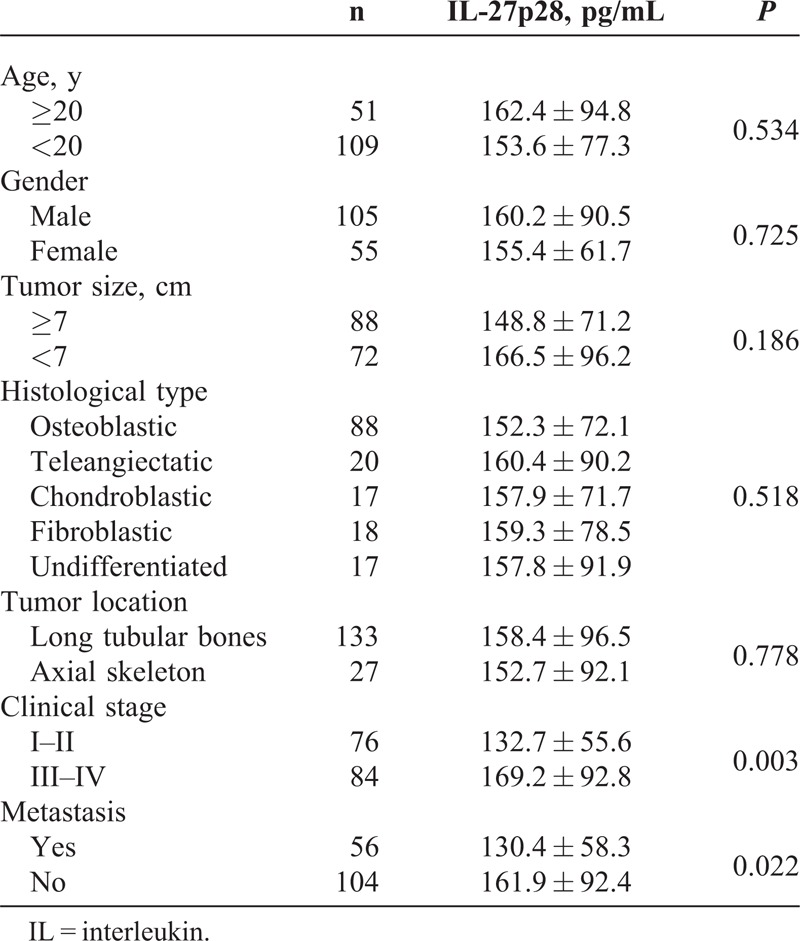
Serum IL-27p28 and Clinical Features of Osteosarcoma

### IL-27 Polymorphisms With Respect to Serum IL-27p28 Levels in Osteosarcoma

No significant differences of the IL-27 gene polymorphisms (-964 A/G, 2905 T/G, and 4730 T/C) were detected with respect to serum IL-27p28 levels in osteosarcoma (see Table 3).

**TABLE 3:**
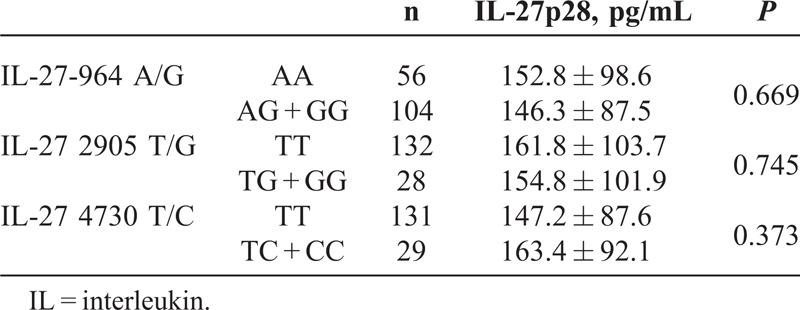
IL-27 Polymorphisms With Respect to Serum IL-27p28 Levels

### Genotype and Allele Frequencies of IL-27 Polymorphisms in Osteosarcoma

The genotype and allele frequencies of IL-27 -964 A/G, 2905 T/G, and 4730 T/C polymorphisms between osteosarcoma patients and controls are shown in Table 4. The genotype distributions of the 3 polymorphisms between the osteosarcoma patients and the controls were all in agreement with HWE (data not shown). However, genotype and allele frequencies of IL-27 -964 A/G, 2905 T/G, and 4730 T/C polymorphisms of osteosarcoma were not associated with osteosarcoma risk (*P* > 0.05).

**TABLE 4:**
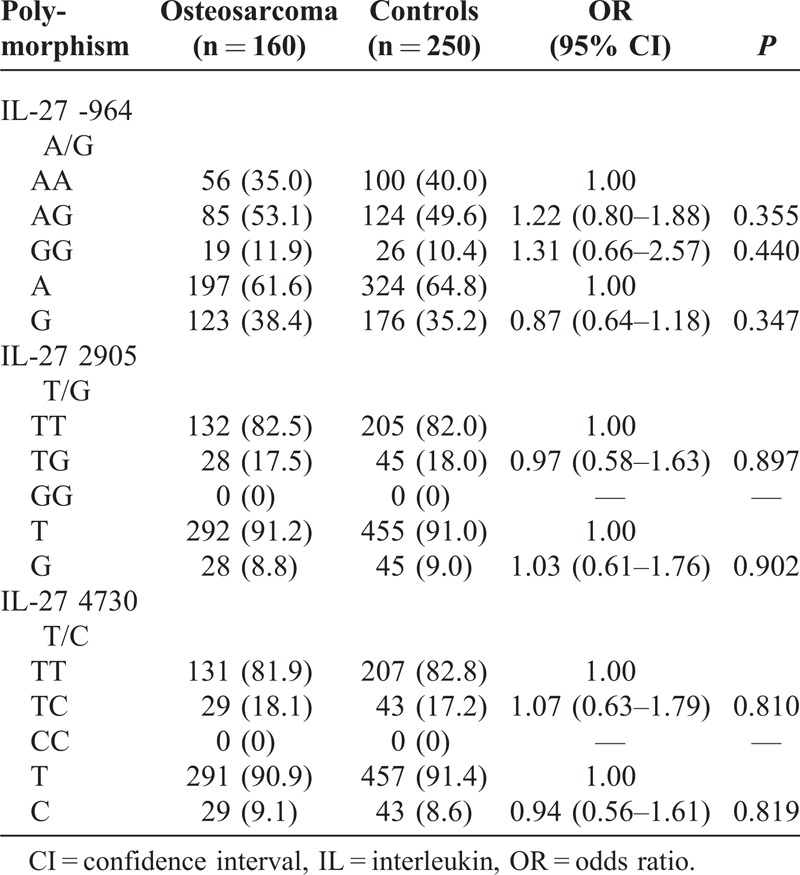
Genotype and Allele Frequencies of IL-27 Polymorphism in Osteosarcoma and Controls

### Distribution of IL-27 Genotype With Clinical Parameters of Osteosarcoma

No associations were found between IL-27 -964 A/G, 2905 T/G, and 4730 T/C polymorphisms and the location, metastasis, and clinical stage of osteosarcoma (*P* > 0.05). See Table S2, http://links.lww.com/MD/A34, which illustrates the distribution of IL-27 genotype with clinical parameters of osteosarcoma.

### Haplotype Analysis of the IL-27 Polymorphisms

Three possible haplotypes were constructed and shown in Table 5 (haplotype frequency <5% was ignored in the table). LD was observed between locus -964 and locus 2905 (|D′| = 0.998), locus -964 and locus 4730 (|D′| = 0.998), and locus 2905 and locus 4730 (|D′| = 0.985). The majority of the haplotypes of osteosarcoma patients and controls accounted for 63.5% and 64.8% of overall haplotypes, respectively. However, none of the IL-27 haplotypes were not associated with osteosarcoma risk (*P* > 0.05).

**TABLE 5:**
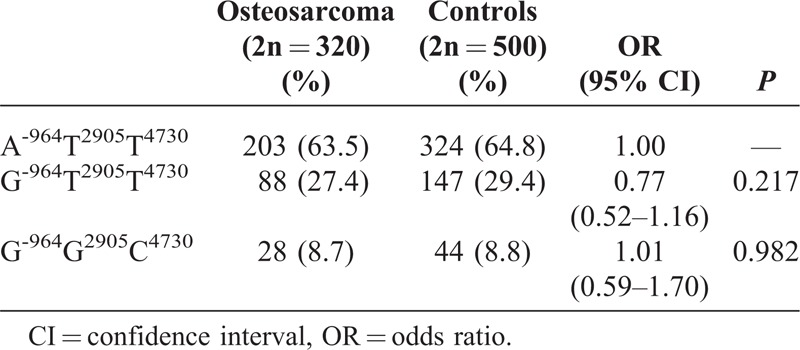
Haplotype Analysis of IL-27 Gene in Osteosarcoma and in Controls

## DISCUSSION

IL-27 is produced by several antigen-presenting cells, such as lipopolysaccharide-stimulated monocytes and monocyte-derived dendritic cells.^18^ The biological functions of IL-27 are mediated by WSX-1 that is highly expressed on CD4^+^ T lymphocytes and natural killer cells.^19^ IL-27 is also known to play multiple roles in the upregulation of Th1 initiation as well as in the downregulation of Th2 factor GATA binding protein 3.^20^ Increased expression of IL-27 is found to be associated with several granulomatous diseases such as tuberculosis, sarcoidosis, and Crohn disease.^21^ Previous studies have indicated that IL-27 was involved in the pathogenesis of several tumors. Feng et al^22^ showed that IL-27 was able to induce the expression of interferon-γ-inducing protein (interferon inducible protein-10/CXCL10) and monokine induced CXCL9 (CXCL9). Both molecules were able to inhibit tumor growth and metastasis through their antiangiogenic effects. Yoshimoto et al^23^ discovered that IL-27 was able to perform direct antiproliferative effects on several WSX-1-positive human melanoma lines. Shimizu et al^24^ indicated that IL-27-transduced tumors have lower microvessel density and grow more slowly than parental tumors. Taken together, the current evidences suggested that IL-27 can inhibit both primary and metastatic tumor, which potentially makes it a valuable therapeutic agent in poorly immunogenic tumors. In the present study, we found that the serum IL-27p28 levels were significantly reduced in osteosarcoma patients compared with healthy controls, suggesting that the low serum IL-27p28 may lead to the development of osteosarcoma by reducing Th1 cytotoxic response.

It is important to note that, besides IL-27, there are other heterodimeric ILs, such as IL-23, IL-12, and IL-35^25^ and both IL-27 and IL-12 are secreted by dendritic cells. Therefore, by determining the serum levels of IL-27 and IL-12, we could know whether the lower levels of IL-27 are related to clinical situations other than osteosarcoma. We found that, in line with the results of IL-27, serum IL-12 levels were lower in osteosarcoma patients compared with healthy controls, and these two cytokines were positively correlated, suggesting that low serum IL-27 levels may be a more general phenomenon in osteosarcoma patients. Next, we investigated the association of IL-27 with clinical features of osteosarcoma patients; the results showed that IL-27 was not related to the patients’ age, gender, tumor size, histological type, and location. Distinct low serum IL-27 levels were presented in osteosarcoma patients of clinical stages III–IV and with tumor metastasis, indicating that low serum IL-27 levels were related to the progression of osteosarcoma.

We noted that the data from previous epidemiological studies in diverse ethnic populations were inconsistent with our results. In the present study, the allele frequencies of the IL-27 -964 G, 2905 G, and 4730 C among the healthy controls were 0.352, 0.090, and 0.086, respectively, which were similar to the frequencies observed in 2 other Chinese reports,^13,16^ but higher than the frequencies observed in healthy Koreans,^10,12^ suggesting that the distribution of IL-27 gene frequencies might vary among different ethnic groups. With respect to the IL-27 polymorphism with serum IL-27p28 levels, although serum IL-27p28 levels were lower in osteosarcoma than in controls, we did not found any differences between serum IL-27p28 levels and IL-27 gene -964 A/G, 2905 T/G, and 4730 T/C polymorphisms, which was similar to what Zhao et al^15^ reported, suggesting that the above 3 variants may not influence the production of IL-27p28 levels.

With regard to IL-27 polymorphisms, previous studies have demonstrated that -964 A/G polymorphism of the IL-27 gene and the haplotypes of -964 A/G, 2905 T/G, and 4730 T/C were associated with the susceptibility to IBD,^12^ asthma,^10^ and COPD.^11^ However, in the setting of tumors, no associations were found with respect to esophageal cancer,^13^ nasopharyngeal carcinoma,^14^ glioma,^15^ and hepatocellular carcinoma.^26^ Our results were in agreement with the results of studies on the tumors. In addition, we also failed to reveal any association between -964 A/G, 2905 T/G, 4730 T/C, and osteosarcoma clinical parameters, suggesting that the genotype and allele of these 3 SNPs did not confer susceptibility to osteosarcoma risk. A plausible explanation of the null association may be because these 3 variants have no influence on the change of serum IL-27p28 levels. Haplotypes often occur when SNPs are located in close physical proximity to each other on the chromosome and are inherited in blocks without recombination between parental chromosomes. It was thought that haplotypes are more powerful for detecting susceptibility alleles than individual polymorphisms.^27^ Therefore, we further performed haplotype analysis to explore the potential associations. However, our haplotype analysis of -964 A/G, 2905 T/G, and 4730 T/C also failed to reveal the association of 3 possible haplotypes with osteosarcoma risk, which is similar to the studies on the glioma^15^ and hepatocellular carcinoma.^26^

To our knowledge, this is the first study to evaluate the association between the SNPs of the IL-27 gene -964 A/G, 2905 T/G, and 4730 T/C polymorphism and the risk of osteosarcoma in a Chinese population. We did not observe any association of IL-27 genotypes, alleles, and haplotypes with osteosarcoma risk. Our results suggest that the IL-27 gene polymorphisms may not be important contributors in the carcinogenesis of osteosarcoma. However, several limitations of the present study need to be noted. First, the number of cases and controls is relatively small, which may undermine the statistical power. Second, our sample is derived from the local region and the genetic background is different from other regions; thus, the conclusion should be interpreted with caution when applying to other ethnicities. Third, the selection of osteosarcoma patients was hospital based, whereas the controls were enrolled from the communities, and the design was case-control design but not random design; thus, a selection bias may occur. Therefore, to further validate the association between IL-27 polymorphism and osteosarcoma risk, a larger sample size of population-bases study with other ethnicities involved is mandatory.

In summary, the present study suggests that low serum IL-27p28 levels may be associated with development and progression of osteosarcoma; however, IL-27 gene polymorphism and their haplotypes may not contribute susceptibility to the osteosarcoma risk.
